# NF-κB fingerprinting reveals heterogeneous NF-κB composition in diffuse large B-cell lymphoma

**DOI:** 10.3389/fonc.2023.1181660

**Published:** 2023-06-02

**Authors:** Eleanor Jayawant, Arran Pack, Heather Clark, Emma Kennedy, Ankur Ghodke, John Jones, Chris Pepper, Andrea Pepper, Simon Mitchell

**Affiliations:** Department of Clinical and Experimental Medicine, Brighton and Sussex Medical School, Brighton, United Kingdom

**Keywords:** systems biology, DLCBL, NFkB, TME (tumor microenvironment), math modeling, lymphoma, computational biology

## Abstract

**Introduction:**

Improving treatments for Diffuse Large B-Cell Lymphoma (DLBCL) is challenged by the vast heterogeneity of the disease. Nuclear factor-κB (NF-κB) is frequently aberrantly activated in DLBCL. Transcriptionally active NF-κB is a dimer containing either RelA, RelB or cRel, but the variability in the composition of NF-κB between and within DLBCL cell populations is not known.

**Results:**

Here we describe a new flow cytometry-based analysis technique termed “NF-κB fingerprinting” and demonstrate its applicability to DLBCL cell lines, DLBCL core-needle biopsy samples, and healthy donor blood samples. We find each of these cell populations has a unique NF-κB fingerprint and that widely used cell-of-origin classifications are inadequate to capture NF-κB heterogeneity in DLBCL. Computational modeling predicts that RelA is a key determinant of response to microenvironmental stimuli, and we experimentally identify substantial variability in RelA between and within ABC-DLBCL cell lines. We find that when we incorporate NF-κB fingerprints and mutational information into computational models we can predict how heterogeneous DLBCL cell populations respond to microenvironmental stimuli, and we validate these predictions experimentally.

**Discussion:**

Our results show that the composition of NF-κB is highly heterogeneous in DLBCL and predictive of how DLBCL cells will respond to microenvironmental stimuli. We find that commonly occurring mutations in the NF-κB signaling pathway reduce DLBCL’s response to microenvironmental stimuli. NF-κB fingerprinting is a widely applicable analysis technique to quantify NF-κB heterogeneity in B cell malignancies that reveals functionally significant differences in NF-κB composition within and between cell populations.

## Introduction

B cell lymphoma is the most common lymphoid malignancy (1). Profiling Diffuse Large B-cell Lymphoma (DLBCL) using gene expression microarray technology revealed two subtypes of the disease, activated B cell (ABC) and germinal-center (GC), which aligned with a distinct cell of origin (COO) (2, 3). More recent molecular subtyping through genomic profiling, in largely simultaneous studies by several groups, found a remarkable agreement on 5-8 genomic subtypes of the disease (2, 4–6). These classifications are prognostically and biologically informative but have not yet translated into stratified treatments (7, 8). Even within these studies there exists substantial heterogeneity in mutational profile, gene expression, and disease outcome suggesting we are still only scratching the surface of the heterogeneity in DLBCL.

In lymphoma, many recurring mutations and stimuli in the tumor microenvironment (TME) converge on Nuclear factor-κB (NF-κB) signaling (9, 10). Within lymph nodes, activated CD4+ T cells express CD40 ligand (CD40L), which activate the non-canonical NF-κB pathway, promoting B cell survival (11). Through this pathway, CD40 activation contributes to oncogenesis and drug-resistance in B cell malignancies (12–14). In DLBCL, CD40 expression is a marker of inflammation in the TME and correlates with improved prognosis but does not correlate with COO subtypes (15, 16). TNFSF13/APRIL secreted by neutrophils in the TME also activates the non-canonical NF-κB pathway in a subset of DLBCL and correlates with a poor prognosis (17). Similarly, BAFF activates the non-canonical NF-κB pathway, and high serum BAFF correlates with poor prognosis in DLBCL patients (18). BCR and TLR signaling also activate the canonical NF-κB pathways in DLBCL. BCR signaling is commonly activated by mutations in ABC-DLBCL, and potentially by autoantigens (19, 20). Microenvironmentally-meditated TLR9 activation signaling to NF-κB has also been shown to promote DLBCL progression (21).

In addition to TME activation, NF-κB is frequently aberrantly activated in lymphoid malignancies through genomic alterations (10, 22–28) and tumor viruses such as EBV (29, 30). Activation of NF-κB in B cells is pro-survival and pro-proliferative (31, 32). Indeed, through combining computational modeling and single-cell experimental analysis, molecular variability in NF-κB signaling has been shown to contribute to variability in proliferation outcomes in B cell populations (32–37). While inhibition of NF-κB can kill DLBCL cells, the ubiquitous role of NF-κB creates severe on-target toxicities, which precludes the clinical use of broad NF-κB inhibitors (38, 39).

NF-κB signaling is not a single pathway mediating the activity of a single transcription factor. In fact, NF-κB signaling activates two distinct pathways (canonical and non-canonical), and NF-κB itself consists of five proteins that can form 15 different dimeric transcription factors (40). Three NF-κB family members (RelA, RelB and cRel) are activators of transcription, whereas two family members (p50 and p52) form heterodimers with the transcriptionally active proteins (41). The most transcriptionally active, well-studied, and relevant NF-κB heterodimers in the context of DLBCL are RelA:p50 and cRel:p50, which are regulated by the canonical pathway, and RelB:p52 which is regulated by the non-canonical pathway (40). While DLBCL research has largely focused on total NF-κB activation resulting from mutations within these pathways, there is growing evidence that the composition of NF-κB within DLBCL is functionally important (42, 43). Canonical pathway activation of both RelA and cRel has been identified, along with non-canonical pathway activation of p52 also occurring in ~25% of DLBCL cases (10, 44, 45). Much of this heterogeneity in NF-κB does not align with known subgroups of DLBCL identified by gene expression or genetic profiling (44). The source of heterogeneity in the NF-κB composition of DLBCL is unknown but likely results from a combination of mutational heterogeneity, epigenetic heterogeneity, and heterogeneity in the microenvironmental stimuli that tumor cells are exposed to. Genetic profiling of DLBCL subtypes found a greater frequency of NF-κB-activating mutations in ABC- as opposed to GC-DLBCL (46, 47). It is unclear whether variability in NF-κB dimer composition can be explained by cell-of-origin, whether variability exists between cell populations of the same cell-of-origin, or whether variability exists even within well-defined DLBCL cell populations such as cell lines. Given the striking molecular heterogeneity that permeates all aspects of DLBCL biology, understanding heterogeneity in NF-κB signaling may unlock pathway- and subunit-specific therapeutic approaches.

Here we profile the heterogeneous state of NF-κB signaling in B cell lymphoma with single-cell resolution to quantify heterogeneity in the composition of NF-κB in DLBCL. We use this data to create computational simulations with single cell resolution, which enable us to predict how mutations impact lymphoma’s heterogeneous response to the TME.

## Materials and methods

### Experimental methods

#### Cell counting

Cell counts and viability were determined by trypan blue exclusion using a 1:1 mixture of cell suspension to 0.4% trypan blue solution (Invitrogen), and a Countess III cell counter (ThermoFisher).

#### Culture conditions for DLBCL cell lines

Three DLBCL cell lines (RIVA, U2932 and HBL-1) were maintained in liquid culture at a density of 0.5 × 10^6^ cells/ml. All cell lines were maintained in complete medium (CM) composed of RPMI (Sigma) supplemented with 10% heat-inactivated FBS (Sigma-Aldrich), 1% L-glutamine (Sigma) and 1% penicillin and streptomycin (Sigma) and were cultured at 37°C in 5% CO_2_ atmospheric conditions.

#### Stimulation of TLR9 signaling with TLR9 agonist

Cell lines were seeded into a 96-well round-bottomed plate at a density of 3 × 10^5^ cells/ml in 150 µL RPMI CM and stimulated for 45 minutes or 2 hours with 1 µM ODN 2006 (Invivogen) at 37°C/5% CO_2_. Following incubation, the contents of each well were transferred to tubes and washed with 1 mL warm Dulbecco’s Phosphate Buffered Saline (PBS; Sigma-Aldrich) for 7 minutes at 350 ×*g*.

#### Processing of DLBCL patient sample

Lymph core biopsy was obtained from a patient attending Eastbourne District General Hospital with informed consent and in accordance with the ethical approval granted to Dr John Jones (REC #: 22/SC/0094). The core was collected into a tube containing RPMI CM, and then transferred into the well of a 6-well plate with 1 mL fresh RPMI CM. The cells were gently disaggregated using a scalpel, and large non-cellular clumps were removed by filtering through a 50 µm mesh. The plate was washed with 1 mL RPMI CM. The cell suspension was centrifuged at 300 ×*g* for 7 minutes, the supernatant was aspirated, and the pellet was resuspended in fresh RPMI CM. Cells were frozen down to -80°C in a CoolCell Container (Corning) at a minimum density of 5 × 10^6^ cells/tube in FBS + 10% DMSO (Sigma-Aldrich) and transferred to liquid nitrogen storage within 24 hours.

Prior to staining, a vial of sample was removed from liquid nitrogen staining and defrosted in a water bath (37°C). Once defrosted, the sample was washed in RPMI CM for 5 minutes at 300 ×*g*. The supernatant was discarded, and the pellet was resuspended in RPMI CM. The cell suspension was passed through a 50 µm mesh to remove aggregated cells, and the cell count and viability was determined.

#### Isolation of human PBMCs from healthy blood

20 mL of blood was collected from a healthy volunteer and the peripheral blood mononuclear cells (PBMCs) were isolated. Blood was layered on top of warm Histopaque (Sigma-Aldrich), and the tube was centrifuged at 900 ×*g* with deceleration set to minimum. The buffy coat layer was carefully removed and transferred to a clean tube and was washed three times in PBS at 350 ×*g* for 5 minutes. The supernatant was aspirated, and the cell pellet was resuspended in in RPMI CM and counted as previously described. The isolated PBMCs were immediately stained as described below.

#### Flow cytometric quantification of NF-κB subunits

Surface and intracellular labeling were performed using a method adapted from Manso and Medina (48). In brief, 1 × 10^7^ cells/mL were washed with an excess of cold Cell Staining Buffer (CSB; BioLegend) at 4°C at 350 ×*g* for 5 minutes. The supernatant was discarded, and the pellet was resuspended in 300 µl of cold CSB, mixed well and incubated for 20 minutes at 4°C to reduce non-specific binding. After incubation, the cell suspension was washed with an excess of cold CSB for 5 minutes at 350 ×*g* and the supernatant aspirated. The cell pellet was resuspended in PBS up to 1 × 10^7^ cells/mL; and 3 × 10^5^ cells (cell lines), 4 × 10^5^ cells (primary DLBCL cells) or 1 × 10^6^ cells (healthy control) were stained per tube in duplicate (antibodies in **Table 1**). Surface labeling was as per the antibody manufacturer’s instructions. Intracellular staining was then performed using the Cyto-Fast Fix/Perm Buffer Set (BioLegend) in accordance with the manufacturer’s instructions. Samples were analyzed by flow cytometry using a CytoFLEX LX flow cytometer (Beckman Coulter).

**Table 1:** Antibody panel for flow cytometry.

Antibody	Conjugate and clone	Supplier
Anti-CD20	Pacific Blue 2H7	BioLegend
Anti-CD38	Brilliant Violet 785 HIT2	BioLegend
Anti-NF-κB p65	APC 14G10A21	BioLegend
Anti-phospho-NF-κB p65 (Ser529)	PE-Cyanine7 B33B4WP	eBioscience
Anti-RelB	Coralite 488 polyclonal	Proteintech
Anti-cRel	PE G-7	Santa Cruz
**Isotype control**	**Conjugate and clone**	**Supplier**
Mouse IgG2a kappa	Pacific Blue MPC-11	BioLegend
Mouse IgG1 kappa	Brilliant Violet 785 MOPC-21	BioLegend
Mouse IgG2b kappa	APC MPC-11	BioLegend
Mouse IgG2a kappa	PE-Cyanine7 eBM2a	eBioscience
Rabbit IgG	Unconjugated* polyclonal	Proteintech
Mouse IgG_1_ kappa	PE	Santa Cruz

*conjugated to CoraLite 488 in-house using FlexAble CoraLite 488 Antibody Labeling Kit for Rabbit IgG (Proteintech).

#### Analysis of flow cytometric data

Gating was performed using a bespoke analysis pipeline developed using Python v3.8 and CytoFlow (https://github.com/cytoflow/cytoflow) or using FlowJo™ v10.8 Software (BD Life Sciences). Initially, samples were gated based on their forward scatter area (FSC-A) *vs.* side scatter area (SSC-A) to select for lymphocytes and remove debris and dead cells. Next, the lymphocyte population was gated based on their forward scatter height (FSC-H) *vs.* FSC-A, to select for single cells. Finally, where there were multiple populations of lymphocytic cells (i.e., primary samples), B cells were gated as a CD20^high^ population.

Histogram plots were prepared using Matplotlib (49) and Seaborn (50). Median fluorescence intensity (MFI) of the B cell population for each fluorophore was calculated. This value was normalized to that of the isotype control value for each fluorophore. Statistical analyses were performed using Python v3.8. Unless otherwise stated, results are presented as mean (+/- standard deviation).

#### Generation of “NF-κB fingerprints”

For each single cell, the isotype control MFI was subtracted from each antibody’s expression value, and data across multiple cell lines was combined and standardized to a *z*-distribution, such that it has a mean of 0 and a standard deviation of 1. Contour plots were generated using Matplotlib (49) and Seaborn (50).

#### Preparation of cell lysates

For each cell line, 8 × 10^6^ cells were counted as previously described, and centrifuged at 1500 rpm for 10 minutes at 4°C. The supernatant was removed, and the pellet was resuspended in ice cold PBS, before being centrifuged again for a further 10 minutes. Ice cold Pierce RIPA buffer (Thermo Scientific) prepared with Halt protease inhibitor cocktail (Thermo Scientific) and 0.5 M EDTA (Thermo Scientific) was added to each cell pellet and incubated on ice for 10 minutes. Each sample was sonicated for 3 cycles (30 seconds on, 30 seconds off) using a Bioruptor Pico sonication device (Diagenode) at 4°C, vortexing after the second cycle. Finally, each sample was centrifuged once more at 14000 rpm for 10 minutes at 4°C to remove any remaining insoluble material and the supernatant was aliquoted. Cell lysates were stored at -80°C until required.

#### Total protein quantification by BCA assay

Total levels of proteins in cell lysates were assessed in duplicate in a 96-well plate using a Pierce BCA Protein Assay Kit (Thermo Scientific) in accordance with the manufacturer’s instructions. Bovine serum albumin standards in triplicate were used to prepare a standard curve of protein concentration (µg/mL) *vs.* absorbance. Protein quantification was performed using a BioTek Synergy HTX multimode reader (Agilent) at 565 nm.

#### Quantification of NF-κB subunits using western blotting

Samples were prepared with 40 µg of protein made up with Bolt LDS sample buffer (Invitrogen) and Bolt sample reducing agent (Invitrogen). Prior to gel electrophoresis, each prepared sample was heated to 70°C for 10 minutes and centrifuged at 10000 rpm for 10 minutes. Gel electrophoresis was performed at 200 V for 30 minutes, using Bolt 4-12% Bis-Tris pre-cast mini gels (Invitrogen) and Bolt MES SDS running buffer (Invitrogen). Proteins were transferred to a PVDF membrane using iBlot Transfer Stacks (Invitrogen) and an iBlot 2 Gel Transfer Device (Invitrogen) according to the manufacturer’s instructions. Following transfer, the membrane was washed in ultrapure water twice and total protein was stained using No-Stain Protein Labeling Reagent (Invitrogen) in accordance with the manufacturer’s recommendations. Total protein was visualized at 600 nm using an Odyssey Fc Imager (Li-Cor).

Detection of proteins of interest was performed using the iBind Flex system (Invitrogen) and incubated for 2.5 hours. Antibodies used are listed in **Table 2**. The membrane was washed in water for 5 minutes and visualized at 700 and 800 nm using the Odyssey Fc Imager. Loading was normalized to total protein stain using Empiria Studio (Li-Cor).

**Table 2:** Antibodies used for western blotting.

Primary antibody	Clone	Supplier	Secondary antibody	Conjugate	Supplier
Anti-NF-κB p65	D14E12	Cell Signaling	Goat anti-Rabbit IgG (H+L)	Alexa Fluor Plus 800	Invitrogen
Anti-RelB	Polyclonal	Proteintech			
Anti-cRel	JM72-93	Invitrogen			

### Computational modeling

Computational model reaction, parameter and rate law tables are available, along with Jupyter Notebooks to generate all computational modeling figures at https://github.com/SiFTW/NFkBModel. Computational models were all created from tables of parameters, reactions and rates laws using bespoke Python code (https://github.com/SiFTW/CSV2JuliaDiffEq), and the generated Julia files that encoded a system of ordinary differential equations was solved using Julia Differential Equations (51, 52). Full details regarding computational modeling methods are provided in the **Supplementary Materials**.

Simulations of cell populations were performed using distributions of parameters representing expression and degradation of modeled molecular species as described previously with 11% coefficient of variance sampled from a normal distribution around the published parameter value truncated at 0 (32). Each cell was run for a steady state phase with fixed input with the final concentrations of the steady state phase being used as input to the time course phase in which dynamic inputs are used (NEMO/IKK activity in the NF-κB model, and TLR activity in the extended model). All simulations were performed in 25 cells except for basal NF-κB fingerprints which were performed in 1000 cells.

Computational models with increased cRel, RelA and RelB were created by increasing the parameter for expression of these proteins by 10-fold. This parameter increase was performed prior to distribution of expression and degradation parameters to introduce cell-to-cell variability, and these increases were maintained throughout the steady state and time course simulation phases. All other parameters were as published (54). To create cell lines based on gene expression values, the expression rate of NF-κB subunits were standardized to zero mean and unit variance, across a library of DLBCL cell lines (53). The expression rate of each NF-κB subunit was scaled using 10^scaling_val^, such that the average cell lines would have the parameter scaled by 10^0^ = 1 (no change) and a cell with expression 1 standard deviation higher than average will have the parameter scaled by 10^1^ = 10 (10-fold increase). Computational models of NF-κB fingerprints were created by manually adjusting the expression of RelA and RelB to recapitulate the experimentally obtained NF-κB fingerprints. MYD88, CD79B and TAK1 mutations were simulated by adjusting the rate of MYD88 self-activation, the basal BCR activation, and the rate of TAK1 activation respectively (see **Supplementary Materials**).

## Results

### Computational modeling predicts that heterogeneous basal expression of NF-κB RelA alters response to microenvironmental stimuli

**Figure 1:**
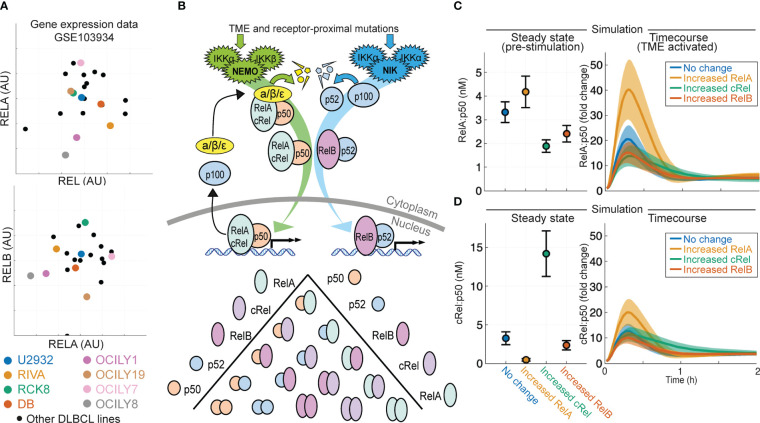
Computational modeling predicts that expression of NF-κB subunit RelA determines response to the tumor microenvironment in DLBCL. **(A)** Expression of RELA (encoding RelA), REL (encoding cRel) and RELB (encoding RelB) in published gene expression data (GSE103934) for a library of DLBCL cell lines (53). Some well-studied cell lines are highlighted with distinct colors. **(B)** Schematic of the scope of the computational model used here (54), which includes both canonical and non-canonical NF-κB signaling and dimer formation between 5 NF-κB component proteins. **(C, D)** Nuclear RelA:p50 **(C)** and cRel:p50 **(D)** concentration in computational simulations using model with no change in parameters, and a 10-fold increase in RelA, cRel and RelB expression. Steady state abundances are shown on the left, with time course responses to TME activation shown on the right. Mean and standard deviation of 25 single cell simulations is indicated.

Analysis of published gene expression data generated on a library of 21 DLBCL lines (53) shows highly heterogeneous and uncorrelated gene expression of NF-κB signaling components cRel, RelA and RelB (gene names REL, RELA and RELB respectively, **Figure 1A**). To predict how heterogeneous expression of these NF-κB transcription factor components within DLBCL might affect the composition of NF-κB dimers and how the disease responds to its microenvironment, we used an established computational model of NF-κB signaling in B cells (54, 56). This model includes both canonical and non-canonical signaling pathways, along with explicitly simulating the many possible NF-κB dimers that can form from the 5 NF-κB monomers (**Figure 1B**). Simulating the impact of an increase in the basal expression of RelA, cRel and RelB on steady state NF-κB composition and the response of NF-κB to TME-mediated canonical pathway activation indicated that the steady-state level of nuclear RelA:p50 would be similar despite these changes (**Figure 1C**, left). Simulations predicted that only increased RelA substantially altered the response to the TME (**Figure 1C**, right). Interestingly, while the model predicted that steady state cRel:p50 may be substantially altered by increased cRel expression (**Figure 1D**, left), the nuclear activity of cRel:p50 in response to the TME is unchanged by large expression changes in NF-κB components (**Figure 1D**, right). These simulations predict that only increased RelA could exceed the inherent cell-to-cell variability within the cell population and substantially alter NF-κB response to the TME (**Figure 1C**).

### Computational models, parameterized by published gene expression data, predict that DLBCL cell lines with the same cell-of-origin can have distinct responses to the TME

**Figure 2:**
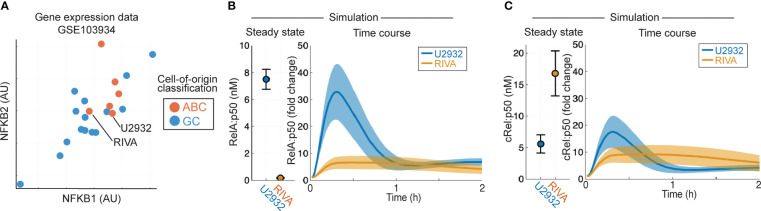
Computational modeling predicts that DLBCL cell lines of the same cell of origin have distinct basal RelA activity. **(A)** Expression of NFKB1 (encoding p105/p50) and NFKB2 (encoding p100/p52) in published gene expression data (GSE103934) for a library of DLBCL cell lines (53). ABC/GC-DLBCL is indicated in red and blue respectively. **(B, C)** Nuclear RelA:p50 **(B)** and cRel:p50 **(C)** concentration in computational simulations using gene expression values to scale the expression of NF-κB components and create cell line-specific models. Steady state abundances are shown on the left, with TME-activated time course responses shown on the right. Mean and standard deviation of 25 single cell simulations is indicated.

Aberrant NF-κB activity is frequently seen in ABC-DLBCL (10), and ABC-DLBCL cell lines have higher expression of NF-κB target genes NFKB1 and NFKB2 in published gene expression data [**Figure 2A**, (53)]. To establish whether heterogeneous gene expression of multiple NF-κB components can confer distinct basal NF-κB activation and response to TME, we simulated the RIVA and U2932 cell lines, which show similar expression of NF-κB target genes and are both ABC-DLBCL cell lines. Published gene expression values were incorporated into the computational model to create cell-line-specific models that were simulated to steady state followed by canonical pathway activation (**Figures 2B, C**). These simulations predict elevated basal nuclear RelA:p50 activity in U2932 cell lines, and an increased response to the TME in U2932 cell lines (**Figure 2B**). Differences in cRel:p50 between these cell lines were predicted to be smaller than differences in RelA:p50 both at steady state and in response to stimuli (**Figures 2B, C**). These simulations predicted heterogeneity in RelA abundance may substantially alter the composition of NF-κB and the sensitivity of DLBCL to the TME, however it is not known whether gene expression heterogeneity is translated to heterogeneity in protein abundance.

### Distinct clonal populations within the U2932 cell line have distinct NF-κB states

**Figure 3:**
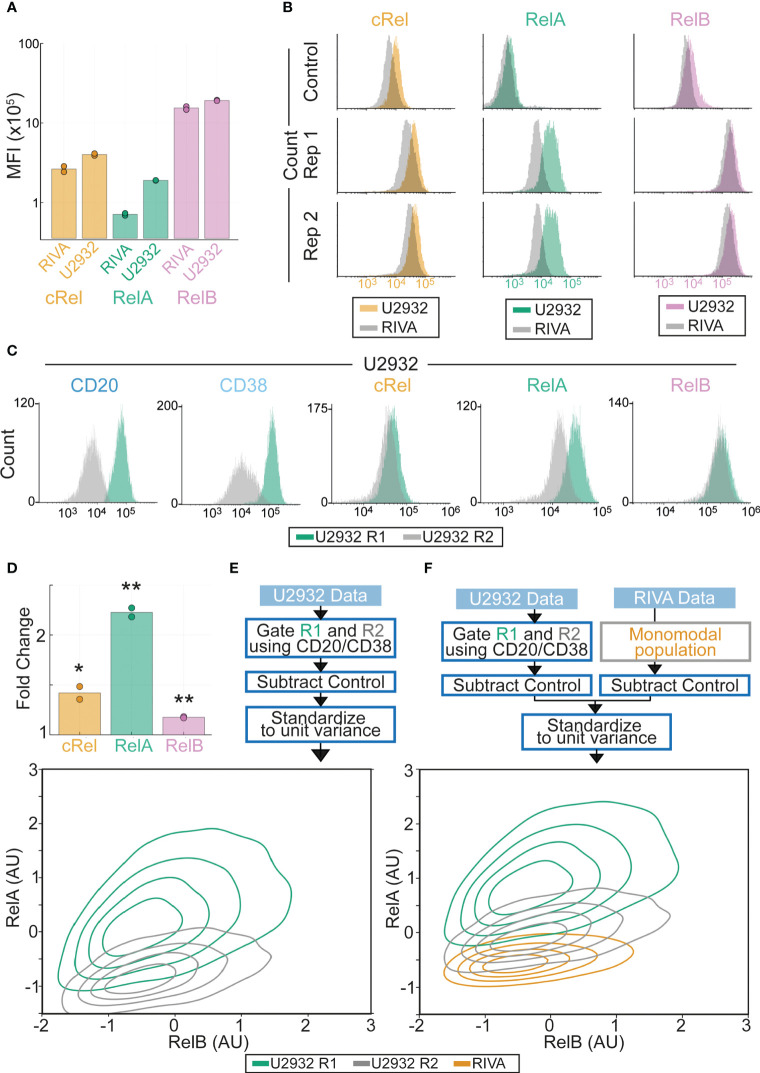
Flow cytometry-based NF-κB fingerprinting reveals unique NF-κB signaling states in subclones of the U2932 cell lines and between ABC-DLBCL cell lines. **(A)** Median Fluorescence Intensity (MFI) of NF-κB components cRel (orange), RelA (green) and RelB (pink) in U2932 and RIVA cell line measured by flow cytometry. **(B)** Histograms of NF-κB components cRel, RelA and RelB in U2932 (color) DLBCL cell line compared to RIVA (gray) DLBCL cell line. Control indicates isotype control. Histograms are normalized to equal peak height. **(C)** Flow cytometry histograms for the indicated proteins in the U2932 R1 and R2 subclones as determined by gating CD38hi/CD20hi compared to CD38lo/CD20lo respectively. **(D)** Fold change in MFI of the indicated NF-κB components between the U2932 R1 and R2 subclones identified as CD20hi (R1) and CD20lo (R2). Mean of two replicates is shown with individual experiments indicated with a dot. * = p<0.05, **=p<0.01 **(E)** NF-κB fingerprinting approach (above) and contour plot (below) for U2932 cell line. Cell density is indicated with a contour plot with the R1 subclone in green and R2 subclone in yellow. **(F)** Same as **(E)** but with the inclusion of data from the RIVA cell line.

As simulations predicted that RelA was a critical NF-κB subunit in determining the TME response of DLBCL cells, and previous studies have implicated both cRel and RelB (44, 57), we sought to establish a flow cytometry-based approach that would enable us to characterize the composition of NF-κB proteins in DLBCL with single cell resolution. A multiparametric flow cytometry panel was established that included the three NF-κB proteins that contain transaction domains, capable of activating gene expression (RelA, cRel and RelB, **Figures 3A, B**). CD20 was included to enable identification of B-lymphocytes in primary samples, and CD38 as a potential surrogate marker for NF-κB activation, as seen in chronic lymphocytic leukemia (58). Given their similarity by cell-of-origin and overall NF-κB activity inferred from gene expression (**Figure 2A**), we first measured the NF-κB composition of the RIVA and U2932 cell lines (**Figures 3A, B**). We found that the two cell lines expressed similar levels of cRel and RelB, but the U2932 cell line contained substantially more RelA (**Figures 3A, B**). While there appeared to be higher cRel in the U2932 cell line compared to RIVA, this was non-specific as demonstrated by a similar difference between the two stained with isotype controls (**Figure 3B**).

**Figure 4:**
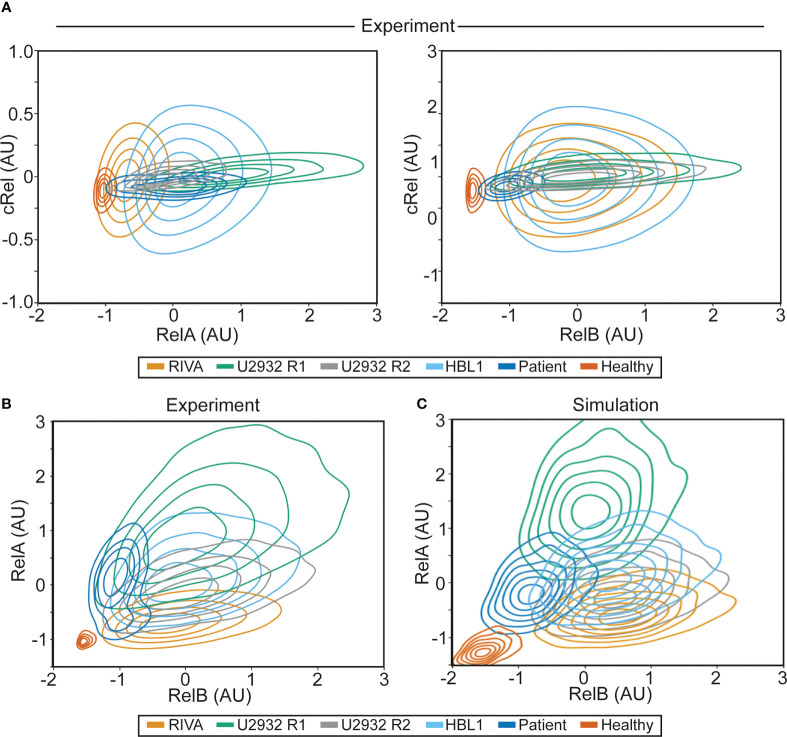
NF-κB fingerprinting can be applied to cell lines, DLBCL patient samples and healthy blood and reveals a unique NF-κB state in each cell population. **(A)** Experimentally measured NF-κB fingerprinting based on RelA and cRel abundance (left), and cRel and RelB abundance (right). Cell density is indicated with a contour plot and each cell population is shown in distinct colors. **(B)** Experimentally measured NF-κB fingerprinting based on RelA and RelB abundance. Cell density is indicated with a contour plot and each cell population is shown in distinct colors. **(C)** Computationally simulated NF-κB fingerprints in six cell population specific computational simulations informed by experimental NF-κB fingerprinting **(B)**. 1,000 cells were simulated in each cell population (6,000 simulations in total), with cell-to-cell variability incorporated as described previously (32), cell density is indicated with a contour plot and each cell population is shown in distinct colors.

The U2932 DLBCL cell line contains two genetically distinct subclones (R1 and R2) that are stably retained and identifiable by differential CD20 and CD38 expression (59)(**Figure 3C**). We found that the CD38^high^ subclone (U2932 R1) had elevated RelA compared to U2932 R2 (**Figures 3C, D**, 2.2 fold, p=0.0014). In this cell line, CD38 activity correlates with increased NF-κB activity. Interestingly, as predicted by computational modeling informed by gene expression data, RelA was found to be significantly heterogeneous between DLBCL cell lines (**Figures 3A, B** and **Figure 1C**). However, flow cytometry revealed additional heterogeneity also exists within the bi-clonal U2932 cell line, which could not be predicted from gene expression data (**Figures 3C, D**).

### Flow cytometry-based NF-κB fingerprinting reveals heterogeneity in the state of NF-κB signaling in DLBCL beyond known subtypes

To overcome challenges in quantitatively comparing flow cytometry results across cell lines we subtracted isotype control MFI values from each antibody’s expression value before combining all the data across cell lines and standardizing the data such that it has zero mean and unit variance. The result was an “NF-κB fingerprint” for a cell population, which allowed multiparametric comparison of the NF-κB status in multiple cell populations while maintaining within-sample heterogeneity (**Figures 3E, F**). This approach allowed quantitative comparison of within-sample and between-sample variability. Combining the U2932 R1 and R2 NF-κB fingerprints data with those from the RIVA cell line revealed that all three cell populations had similar expression of all measured NF-κB components except for substantial differences in RelA, with the lowest RelA expression in RIVA cells (**Figure 3F**). Western blots for RelA, RelB and cRel confirmed that RelA was substantially higher in the U2932 cell line than the RIVA cell line, with the cRel and RelB expression being similar between these two cell lines (**Figure S1**). These distinct NF-κB fingerprints despite similar gene expression profiles and the same cell-of-origin, highlight that cell-of-origin is insufficient to describe the heterogeneity in NF-κB state between DLBCL lines. We find that while cell-to-cell variability results in overlapping NF-κB fingerprints, between sample variability in RelA results in distinct NF-κB fingerprints, even within subclones of the same cell line (**Figure 3F**).

### NF-κB fingerprinting reveals substantial NF-κB heterogeneity within and across cell lines, primary DLBCL and healthy B cells

To establish whether the heterogeneity observed in RelA (but not RelB or cRel) between RIVA and U2932 cell lines was a property of ABC-DLBCL cell lines, all DLBCL cells or all B cells, we performed further NF-κB fingerprinting on another ABC-DLBCL cell line (HBL-1), a primary DLBCL patient lymph node biopsy, and healthy primary B cells extracted from peripheral blood (**Figure 4**). We were able to generate NF-κB fingerprints for all these sample types. Variability in cRel was all explained by within-sample cell-to-cell variability, with no variability between samples. The extent of variability within a sample differed between cell lines, with the RIVA and HBL1 cell lines showing the highest cell-to-cell variability in cRel expression (**Figure 4A**). The primary DLBCL patient B cells and healthy donor B cells had the lowest cRel variability (**Figure 4A**). While all the cell lines showed similar RelB expression, dominated by within-sample variability, the primary B cells both showed lower expression of RelB with less within-sample variability (**Figure 4A**).

As the largest variability between cell populations was seen in RelA and RelB expression, plotting NF-κB fingerprints based on RelA and RelB expression revealed distinct NF-κB fingerprints in each of the measured cell populations (**Figure 4B**). The ABC-DLBCL line HBL1 expressed similar RelB and cRel, consistent with the other cell lines assayed, and similar RelA expression to U2932 cells, placing its fingerprint between U2932 R1 and R2 subclones (**Figure 4B**). Taken together, all the ABC-DLBCL cell lines (and clonal populations within cell lines) differ in their expression of RelA alone (**Figure 4B**). Interestingly, this is the expression change that simulations predict to be the most impactful on response to the TME, suggesting these cell lines may have distinct responses to the TME (**Figure 1C**).

To determine whether these distinct NF-κB fingerprints may modulate the TME, in addition to modulating the cellular response to TME stimuli, we analyzed published RNA-sequencing data across a library of DLBCL cell lines. This analysis revealed that cell lines with high expression of RelA also had higher expression of multiple immunomodulatory cytokines and chemokines (**Figure S2**). The same was not true of cRel, or RelB, and no difference was seen between cell lines stratified by COO (**Figures S2, S3**). Combining our computational modeling with these gene expression data suggests that RelA-high DLBCL cells may create a more inflammatory TME, while also amplifying their response to this inflammatory microenvironment (**Figures 1C** and **Figure S2**).

Performing NF-κB fingerprinting on a diagnostic DLBCL lymph node biopsy also revealed a unique fingerprint with substantial within-sample heterogeneity in RelA that spanned the cell lines profiled (**Figure 4B**). The difference in RelA expression between the major and minor cellular sub-population in the patient sample is consistent with the difference in RelA expression within the U2932 subclones (**Figure 4B**). While the primary DLBCL sample was homogeneous for RelB and cRel, its fingerprint did reveal distinct RelB expression from the cell lines assayed. The NF-κB fingerprint of healthy B cells was also found to be distinct from all DLBCL cells profiled, with strikingly homogeneous and low expression of both RelB and RelA (**Figure 4B**). Taken together, we found that NF-κB fingerprinting can be applied to a variety of cellular sources and uncovers striking heterogeneity in RelA even between cell lines with the same COO, and within a single patient sample.

### Computational modeling can recapitulate distinct NF-κB fingerprints in DLBC

Multiple sources of heterogeneity can explain within- and between-sample variability. These sources of variability include genetic heterogeneity, epigenetic heterogeneity, molecular variability that can be inherited across cell division, and inherently stochastic processes such as noisy gene expression through “transcriptional bursting” (33). Studies that combined computational modeling with single cell lineage tracking established that cell-to-cell variability within a B cell population is predominantly explained by non-genetic molecular variability. This variability may be accumulated over many rounds of imperfect inheritance of molecular network components during cellular proliferation (32, 33, 35). Therefore, we hypothesized that the same source of variability could explain within-sample cell-to-cell variability in the measured NF-κB fingerprints. To test this hypothesis, we created cell-population-specific computational models of NF-κB signaling by altering the expression of RelA and RelB as determined by experimental NF-κB fingerprinting, and assumed cell-to-cell variability within a cell population was consistent with cell-to-cell variability in non-malignant activated B cells (11% coefficient of variance introduced to parameters that control expression and degradation of molecular components). The resulting cell-line/population-specific models had strikingly similar NF-κB fingerprints to the experimental results (**Figure 4C**). This indicates that the same inherent molecular variability seen in non-malignant B cells explains cell-to-cell variability within mono-clonal B-cell populations. Interestingly, simulated healthy donor B cells over-estimate the cell-to-cell variability, indicating that resting cells have low cell-to-cell variability, while proliferating B cells, whether through malignant transformation or immunogenic activation, have high cell-to-cell variability. As NF-κB fingerprinting only measures total protein content of each cell, we could not determine the overall level of activation of each cell. We found that computational models with high or low NEMO-IKK activity could be indistinguishable using NF-κB fingerprints alone (**Figure S4** and **Figure 4C**). As activating mutations in each cell line likely alter the basal level of NEMO-IKK, we hypothesized that NF-κB activating mutations combine with NF-κB composition to control the cellular response to TME stimuli.

**Figure 5:**
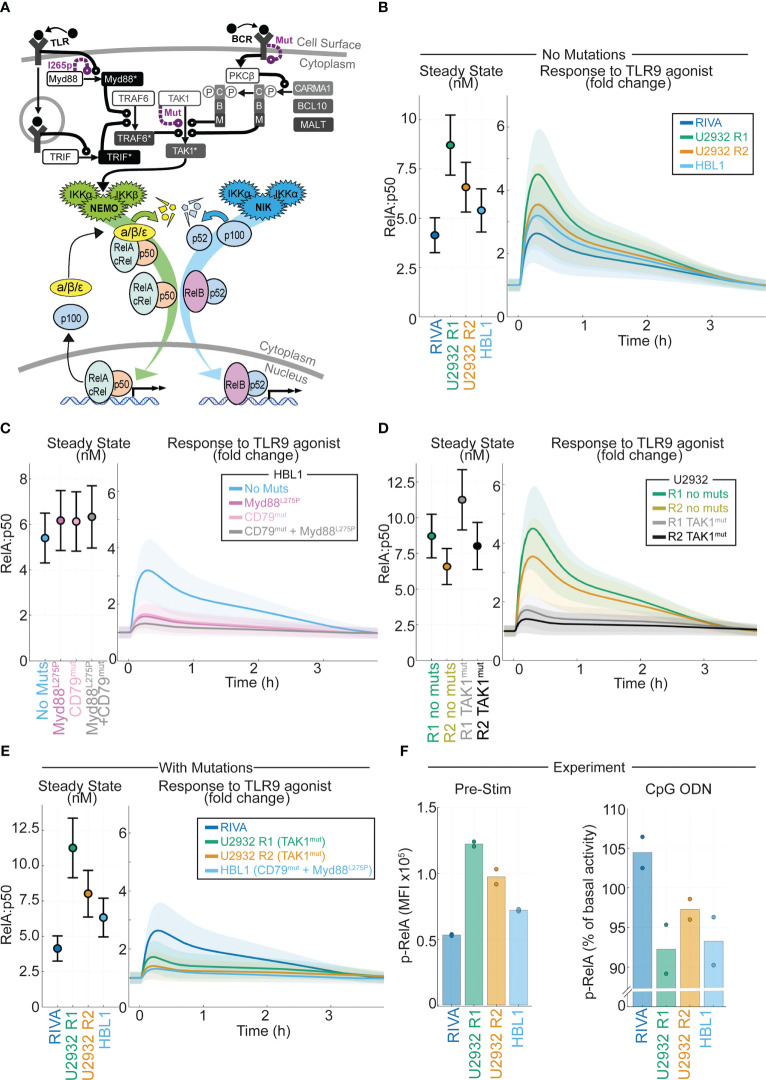
Computational modeling of DLBCL, including receptor-proximal signaling, enables integration of NF-kB fingerprints and mutational data to predict response to the tumor microenvironment. **(A)** Schematic of the computational model constructed by combining existing models of TLR signaling (55), BCR signaling (37), and NF-kB/IkB regulation (54). All models are run as published with active IKK species summed from the BCR and TLR models to determine the active IKK input curve to the NF-kB model. Schematic combines some repeated species, a more detailed schematic is included in Figure S3. **(B)** Cell line specific simulations of nuclear RelA:p50 in each virtual cell line at steady state (left) and the fold change in nuclear RelA:p50 in response to stimuli (right). Mean and standard deviation of 25 single cell simulations is indicated. **(C)** Simulated abundance of nuclear RelA:p50 in virtual HBL1 cell line with no changes (blue), with auto-activating Myd88 to recapitulate MYD88l265p (purple), and with high basal BCR signaling to recapitulate CD79B mutations present in this cell line (pink), and the combination of the two mutations (grey). Mean and standard deviation of 25 single cell simulations is indicated. **(D)** Simulated nuclear RelA:p50 in virtual U2932 cell line with no changes (R1 green, R2 yellow), with increased TAK1 activity recapitulate the TAK1 mutation present in this cell line (R1 grey, R2 black). Mean and standard deviation of 25 single cell simulations is indicated. **(E)** Cell line specific simulations of nuclear RelA:p50 in each virtual cell line at steady state (left) and the fold change in nuclear RelA:p50 in response to stimuli (right), with mutational event included from panels **(C, D)**. Mean and standard deviation of 25 single cell simulations is indicated. **(F)** Experimentally measured median fluorescence intensity (MFI) of phosphorylated RelA in each indicated cell line. The mean of two replicates is shown with individual experiments indicated with a dot. The unstimulated MFI is shown (left) with the percentage change in MFI following 45 mins of activation of TLR9 with CpG ODN shown (right).

### Computational modeling predicts NF-κB fingerprints correlate with TME responses in DLBCL cell lines

In DLBCL, mutations in NF-κB signaling do not frequently occur in the core signaling network of NF-κB-IκB signaling, but occur in the molecular network that transduces receptor-proximal signaling to NEMO-IKKα/β and NIK-IKKα (10). We incorporated these molecular networks into our computational modeling by integrating published models of TLR and BCR signaling, converging on NEMO-IKK, with the model of core NF-κB signaling (37, 55). The resulting combined model’s scope includes many genes that are commonly mutated in DLBCL including MYD88, CD79B and TAK1 (**Figure 5A** and **Figure S5**). Using the simulated DLBCL cell lines (**Figure S1**), we simulated the response to TLR9 activation in the TME (21). As expected from previous simulations (**Figure 1C**), the cell lines with increased RelA expression are predicted to display increased activation of RelA in response to TLR9 activation (**Figure 5B**).

### Incorporating mutations and NF-κB fingerprints into computational models accurately predicts heterogeneous response to the TME

Recurrent mutations occur in DLBCL that are likely to disrupt the signal transduction between the TME and NF-κB (10). Some of these mutations exist in the cell lines studied here, and we hypothesized that they may alter the predicted response to the TME. The HBL1 cell line has both MYD88^l265p^ and CD79B activating mutations (60). Introducing Myd88 mutations (by increasing Myd88 self-activation rate) or CD79B mutations (increasing the level of chronic BCR activation) to the HBL1 specific model does not increase basal nuclear RelA:p50 activation (**Figure 5C** left). However, each of these mutations substantially reduces the induction of nuclear RelA:p50 in response to TLR activation (**Figure 5C** right). The combination of mutations reduces activation to within the standard deviation of inherent cell-to-cell variability in the unstimulated cell population, effectively entirely abrogating activation of RelA:p50 in response to the TME (**Figure 5C** right). The U2932 cell line has TAK1 mutations (10). Simulating the impact of this mutation predicts that this mutation does not increase basal nuclear RelA:p50, but does substantially reduce the activation of RelA in response to TLR activation (**Figure 5D**). The RIVA cell line expresses wild type Myd88, TAK1 and CD79 genes (61). When we compare simulations informed by NF-κB fingerprinting alone (**Figure 5B**) with simulations that also consider mutations (**Figure 5E**) we find that the basal steady-state NF-κB activity is unchanged. However, mutations present in these cell lines mean that cells with increased RelA expression no longer have increased RelA:p50 response to a simulated TME stimulus. In fact, we find that all our cell lines are expected to be broadly unresponsive with only the RIVA line predicted to significantly respond to the TME once mutations are considered. To confirm this prediction, we experimentally measured phosphorylated-RelA, a marker of RelA activity, in the basal state and 45 minutes after exposure to a TLR9 agonist. In line with computational predictions, the highest RelA activity in the basal state was found in U2932 R1 cells with the lowest in RIVA cells (**Figure 5F** left). In response to TLR9 activation, we found only RIVA cells upregulated their RelA activity (**Figure 5F** right). Taken together this demonstrates that incorporating mutational information into computational models, in addition to NF-κB fingerprint information, enables accurate prediction of heterogeneous response to the TME in DLBCL.

## Discussion

Here we develop a new approach to quantifying the NF-κB composition of B cells: NF-κB fingerprinting. We apply NF-κB fingerprinting to DLBCL cell populations, and use this data iteratively, in combination with computational modeling, to reveal striking diversity in the NF-κB state of DLBCL. While previous studies have indicated that NF-κB activation is primarily restricted to ABC-DLBCL (10), more recent work has implicated NF-κB cRel in GC-DLBCL (57). Computational modeling suggested that basal expression of NF-κB RelA is the most important factor in determining how NF-κB responds to the TME. These simulations also predicted that increased RelA activity can decrease cRel activity through competition for p50. We found strikingly heterogeneous RelA expression between cell lines of the same COO and even within a single ABC-DLBCL cell line. This study, combined with recent work that identified RelB activation in another subset of DLBCL that also did not align with COO, indicating that this is insufficient to characterize the heterogeneity of NF-κB in DLBCL (44).

Due to the inclusion of cell surface markers that enable the identification of B cells in blood, NF-κB fingerprinting is a useful tool for quantifying the NF-κB state of B cells within primary samples. Here we find we can apply NF-κB fingerprinting to cell lines, needle core biopsies from DLBCL patients, and blood samples from healthy donors. Strikingly, we found unique NF-κB fingerprints in every cell population tested with distinct NF-κB fingerprints between healthy and malignant B cells.

Many potential sources of variability can contribute to the NF-κB state of a DLBCL cell and its response to the TME, including: mutational heterogeneity, stochastic gene expression noise, distinct epigenetic states, and inheritable molecular variability that accumulates over many generations of cell division. To characterize cell type-specific responses to TME stimuli, here we used DLBCL cell lines as model systems. Such cell lines are a useful tool to study the TME, as they have well-characterized genetic backgrounds, and do not require microenvironmental support for survival, but can still be modulated by TME stimuli. Primary cells, on the other hand, require complex co-culture conditions to allow for survival long enough to perform experimental manipulation, which would confound the effect of TME stimuli (62). Future studies will assess whether combining computational modeling and NF-κB fingerprinting can provide insight into how mutations and the TME together affect NF-κB in DLBCL patient samples.

Due to the single-cell resolution of flow cytometry, NF-κB fingerprints are valuable for informing computational modeling, and combining the approaches provides insight into the different sources of variability in response to the TME. Previous work found that distinct expression of NF-κB components between cell types, potentially through cell type-specific epigenetic states, could explain cell type-specific responses to stimuli (54). The variability in RelA and RelB expression we find here, in the absence of mutations directly affecting these genes, is consistent with distinct epigenetic states between cell populations, but further work is required to measure this directly.

Single-cell lineage tracking combined with computational modeling has also been used to quantify molecular variability that accumulates as B cells divide and imperfectly inherit the molecular components of the cell (32). We found that the same magnitude of inherent variability captures within-sample variability in NF-κB components in DLBCL cell populations and explains the cell-to-cell variability in response to the microenvironment. To summarize, we find that cell-to-cell variability in NF-κB is consistent with the inherent molecular variability found in primary non-malignant B cells, while between cell populations there exists distinct expression of NF-κB components RelA and RelB that primes DLBCL cells to have distinct responses to the TME.

Here we created a new computational model that combines existing models of BCR and TLR receptor-proximal signaling and a comprehensive multi-dimer model of core NF-κB signaling (37, 55, 56). There are very rarely mutations in the genes encoding the five constituent NF-κB proteins [REL, RELA, NFKB1, NFKB2 and RELB are mutated in 0.7%, 0%, 0.7%, 2.2% and 1.5% of DLBCL cases respectively (5)]. Instead, recurrent mutations in DLBCL occur in upstream signaling pathways. As these pathways are also responsible for transducing microenvironmental signals, this new model is a valuable tool for integrating mutational information with signaling states to understand NF-κB regulation in DLBCL. Here we find that only by integrating both signaling and genetic information in this computational framework can we predict how DLBCL will respond to the TME. As the expression of immunomodulatory cytokines was found to be strongly related to RelA activity, and the response to TME stimuli also related to RelA activity. This creates a potential feedback loop in which high RelA creates a more inflammatory microenvironment while also modulating how DLBCL cells respond to the microenvironment. Our investigations were restricted to activation of the canonical NF-κB pathway, however the model presented here provides a tool for future work investigating the role of non-canonical pathway activation in the TME through CD40 or BAFF, both of which have been found to be prognostically significant in DLBCL (16, 18, 63). Given the complex and dynamic regulation between NF-κB and pathways controlling B cell proliferation, apoptosis and terminal differentiation it is likely that incorporating the model we assembled here into larger multi-scale modeling frameworks will provide an opportunity to interrogate how multiple mutations combine in DLBCL, and how to overcome this dysregulation (32, 34). We expect the striking heterogeneity observed here to be seen between DLBCL patients, and that this heterogeneity will not align with clusters from molecular subtyping of DLBCL.

Historically, the use of NF-κB inhibitors in the clinic has been largely unsuccessful due to severe on-target toxicities (38, 39). However, there has been recent rapid progress in the development of small molecule inhibitors and proteolysis targeting chimeras (PROTACs) which target specific NF-κB pathways or components (64–66), likely with reduced toxicity compared to broad-targeting NF-κB inhibitors. Given the heterogeneity of NF-κB signaling in DLBCL, improving treatments in hematological malignancies using these next-generation targeted NF-κB inhibitors will require identification and targeting of a particular subunit or pathway in each specific patient. While NF-κB remains a promising target for the treatment of DLBCL we expect this more personalized approach will be required, and computational simulations together with NF-κB fingerprinting provide a valuable framework for personalized treatment predictions.

## Data availability statement

The datasets presented in this study can be found in online repositories. The names of the repository/repositories and accession number(s) can be found in the article/**Supplementary Material**.

## Ethics statement

The studies involving human participants were reviewed and approved by NHS Health Research Authority. The patients/participants provided their written informed consent to participate in this study.

## Author contributions

EJ, ArP, AG, and SM performed computational analysis and modeling. EJ, HC, EK, JJ, CP, and AnP performed laboratory work. JJ provided access to patient samples. EJ, ArP, SM, CP, and AnP conceptualized the study. All authors wrote and edited the manuscript. JJ, CP, AnP, and SM supervised the project. All authors contributed to the article and approved the submitted version.
