# Supplementary material for: NF-κB fingerprinting reveals heterogeneous NF-κB composition in diffuse large B-cell lymphoma
Source: Front Oncol. 2023 Jun 2;13:1181660. doi: 10.3389/fonc.2023.1181660 (PMC10272839; doi:10.3389/fonc.2023.1181660)
Supplement: Supplementary file 1 [file DataSheet_1.pdf]

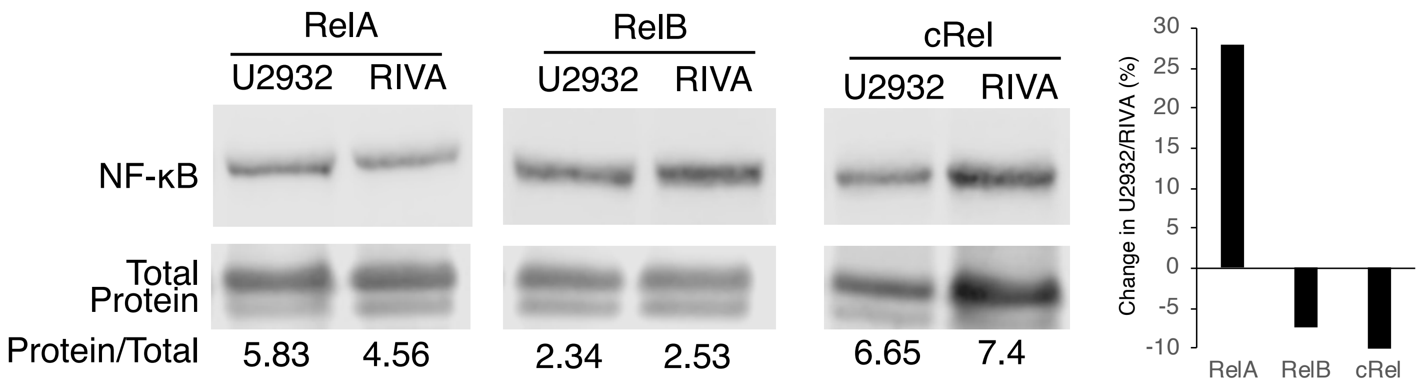

Figure S1. Western bot quantification of NF-κB subunits in U2932 and RIVA cell lines. Each band is quantified relative to total protein loading (bottom). The change in expression in the U2932 cell line normalised to the expression in RIVA is displayed as a percentage (right).

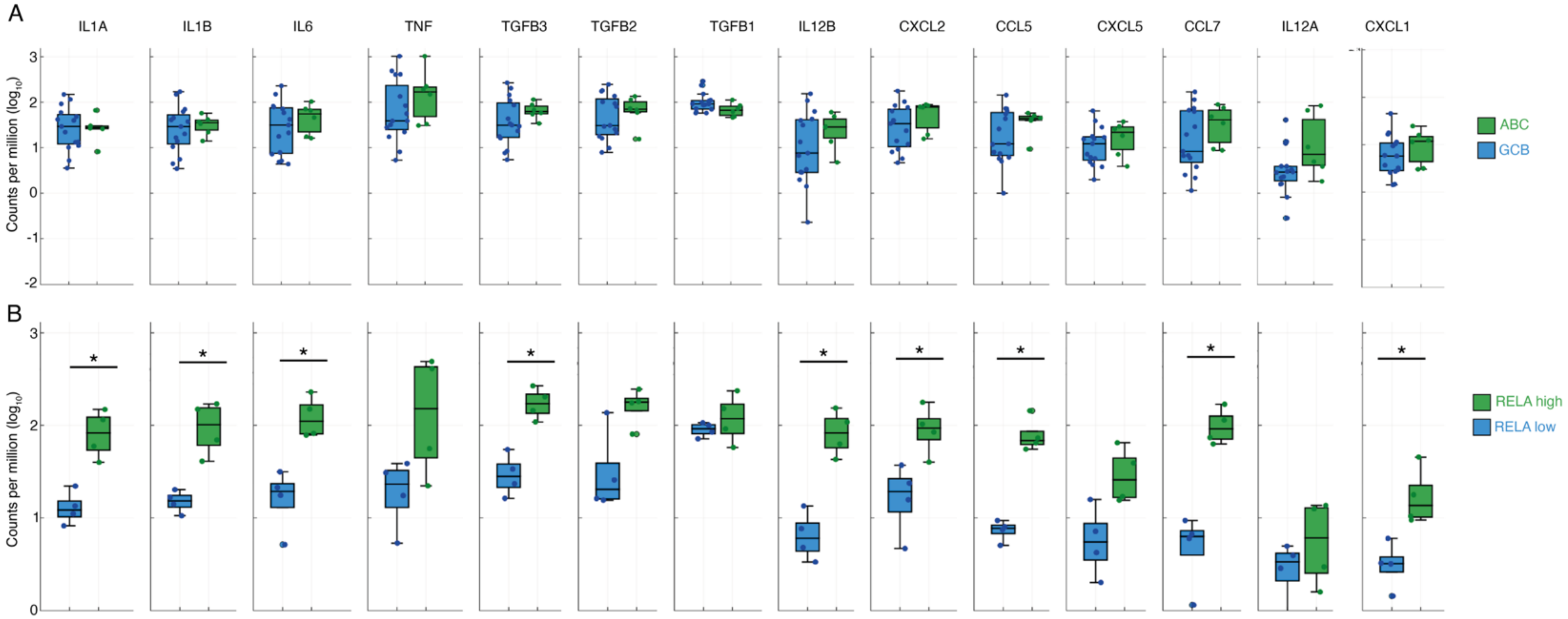

Figure S2. Quantification of published gene expression data (GSE103934) from a library of DLBCL cell lines (1). Expression of the indicated cytokine/chemokine is stratified by cell of origin (A) and RelA expression (B). RelA high = top 20%, and RelA low = bottom 20% of RelA expression. Box plots show mean and interquartile range with whiskers indicating the range of the data excluding outliers. * = P<0.05 by Kolmogorov–Smirnov (KS) test.

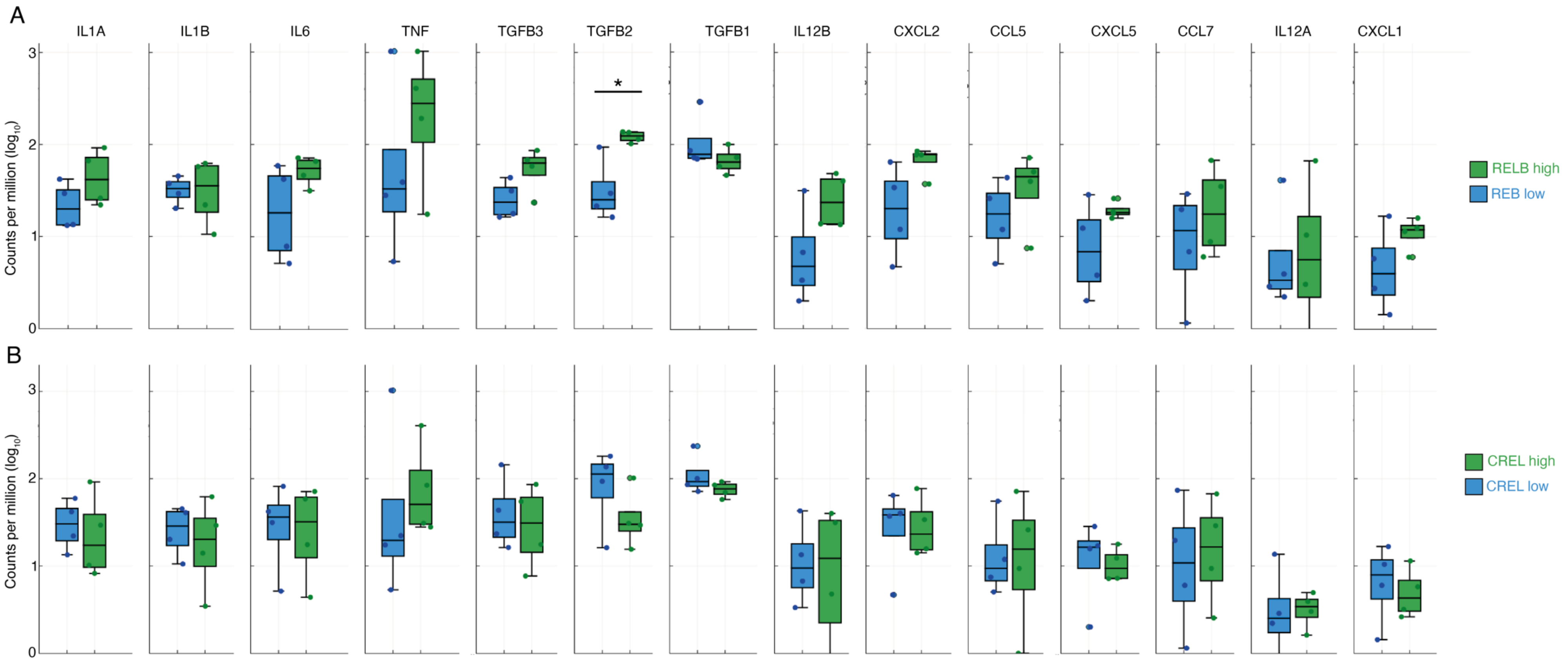

Figure S3. Quantification of published gene expression data (GSE103934) from a library of DLBCL cell lines (1). Expression of the indicated cytokine/chemokine is stratified by RELB (A) and REL (cRel) expression (B). REL/RELB high = top 20%, and REL/RELB low = bottom 20% of RelA expression. Box plots show mean and interquartile range with whiskers indicating the range of the data excluding outliers. * = P<0.05 by Kolmogorov–Smirnov (KS) test.

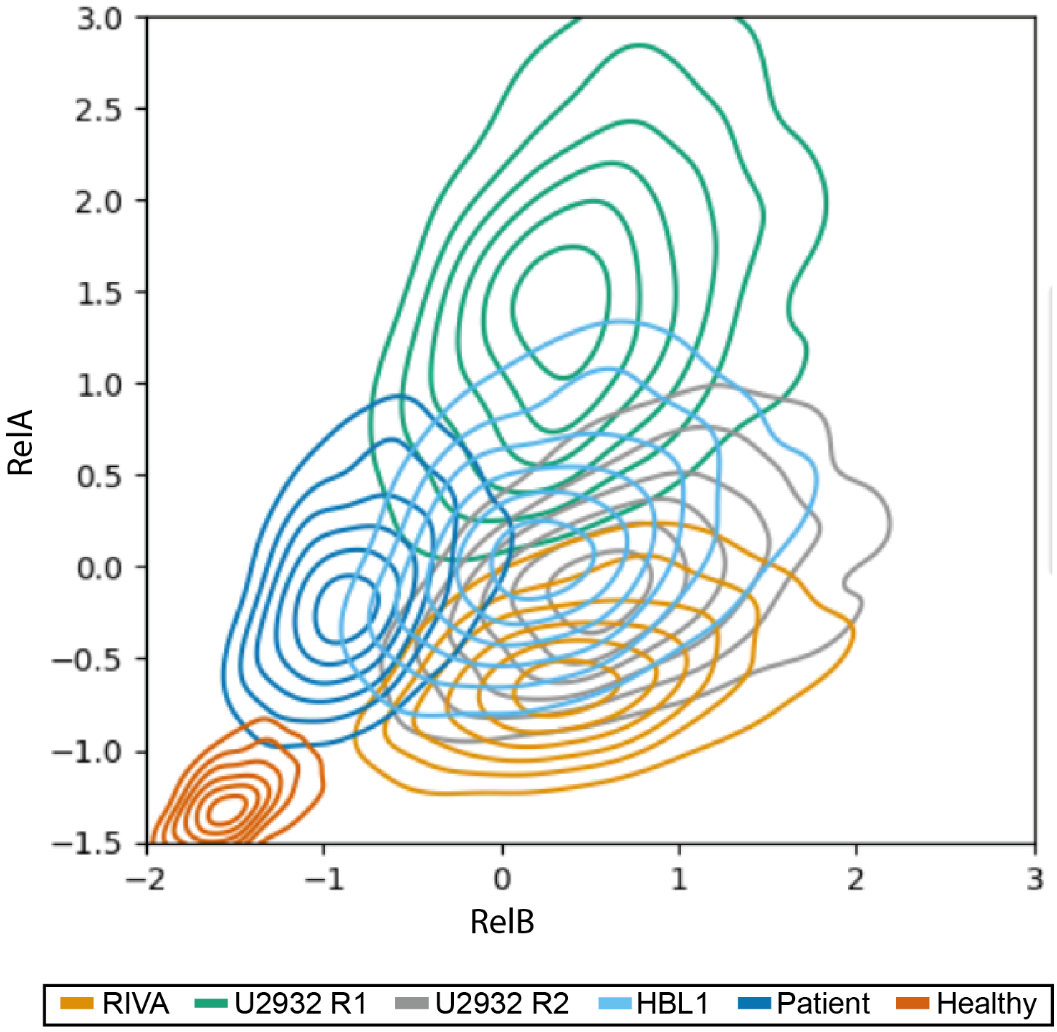

Figure S4. NF-κB activation state cannot be determined from NF-κB fingerprints. Computationally simulated NF-κB fingerprints in six cell population specific computational simulations informed by experimental NF-κB fingerprinting (Figure 4B). Simulation are the same here as in Figure 4C, except for the U2932 cell line where basal IKK activity was increased 100x and the expression of RelA and RelB increased to recapitulate experiment fingerprint (Figure 4B). 1,000 cells were simulated in each cell population (6,000 simulations in total), with cell-to-cell variability incorporated as described previously (2), cell density is indicated with a contour plot and each cell population is shown in distinct colors.

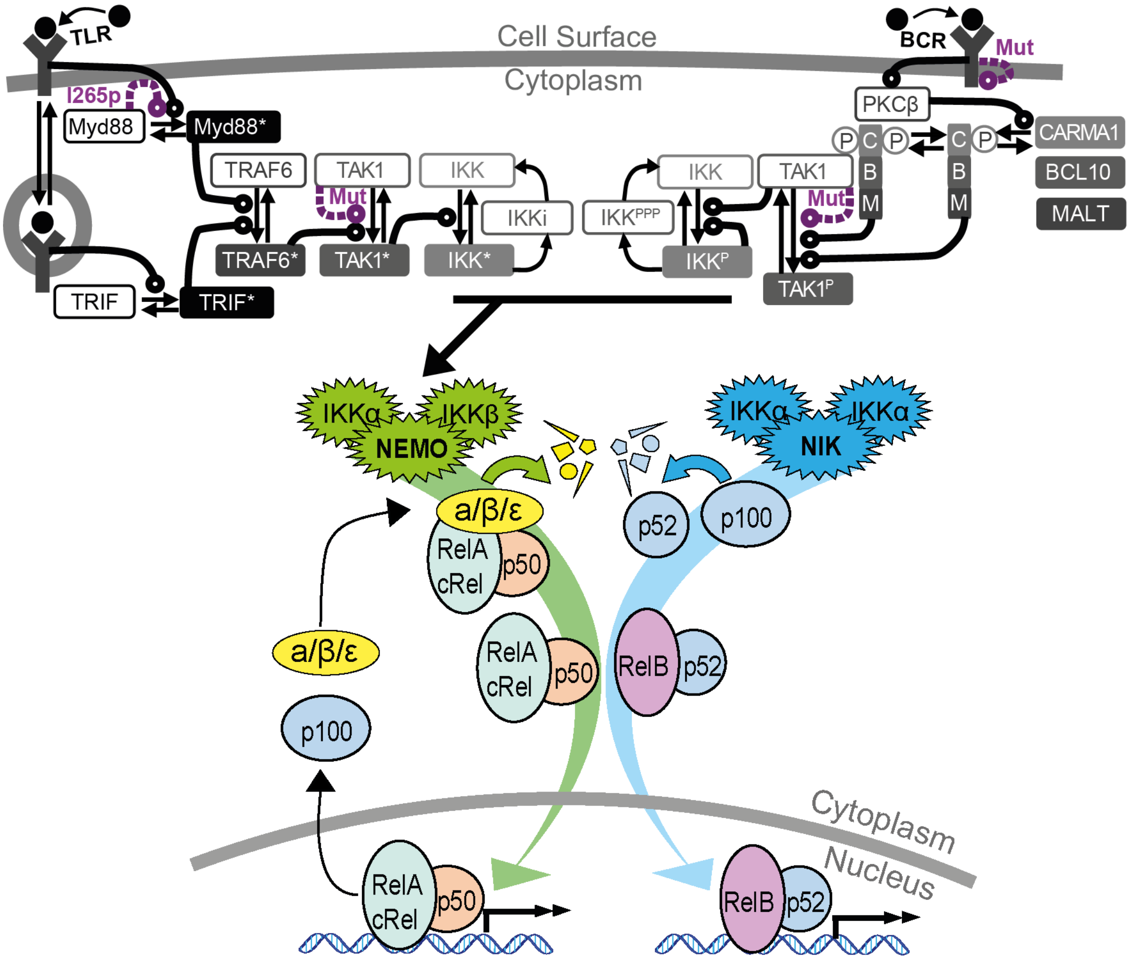

Figure S5. Computational modeling of DLBCL, including receptor-proximal signaling. **A)** Schematic of the computational model constructed by combining existing models of TLR signaling (3), BCR signaling (4), and NF-κB/IκB regulation (5). All models are run as published, with active IKK species summed from the BCR and TLR models to determine the active IKK input curve to the NF-κB model. Mutations present in DLBCL are indicated in purple. Detail of the combinatorial complexity of NF-κB dimer and inhibitor interactions are omitted.

Supplemental Modeling Methodology

All modeling files are available at https://github.com/SiFTW/NFkBModel. This repository includes the Jupyter notebooks for running the models and producing the figures.

Software and versions
Computational simulations were performed using the free DifferentialEquations.jl package, in the free Julia programming language (version 1.6.2). The version number for each package used in this study is provided below:
[fbb218c0] BSON v0.3.6
[336ed68f] CSV v0.10.8
[a93c6f00] DataFrames v0.22.7
[2b5f629d] DiffEqBase v6.84.0
[0c46a032] DifferentialEquations v6.20.0
[31c24e10] Distributions v0.25.45
[5789e2e9] FileIO v1.16.0
[6a86dc24] FiniteDiff v2.17.0
[28b8d3ca] GR v0.64.4
[09f84164] HypothesisTests v0.10.11
[7073ff75] IJulia v1.23.3 `https://github.com/JuliaLang/IJulia.jl.git#master`
[033835bb] JLD2 v0.4.3
[b964fa9f] LaTeXStrings v1.3.0
[b4fcebef] Lasso v0.6.2
[21d151f5] LassoPlot v1.1.1
[1dea7af3] OrdinaryDiffEq v5.71.2
[69de0a69] Parsers v2.2.4
[58dd65bb] Plotly v0.3.0
[a03496cd] PlotlyBase v0.5.4
[f0f68f2c] PlotlyJS v0.14.1
[91a5bcdd] Plots v1.32.0
[438e738f] PyCall v1.94.1
[d330b81b] PyPlot v2.11.0
[1a8c2f83] Query v1.0.0
[102930c3] SmoothingSplines v0.3.1
[f3b207a7] StatsPlots v0.14.34
[c3572dad] Sundials v4.11.4
[bd369af6] Tables v1.10.0
[a759f4b9] TimerOutputs v0.5.22
[0f1e0344] WebIO v0.8.20

Generating models from reactions, parameters and rate laws.
Computational models were encoded across three .csv files: reactions.csv, parameters.csv, and rateLaws.csv (available on GitHub https://github.com/SiFTW/NFkBModel). All parameters in all simulations were kept consistent with published parameters (5), with the exception of cell line-specific parameterizations defined below. Bespoke Python 3 code was used to assemble these files into a single Julia (.jl) file of equations written into a function compatible

with the DifferentialEquations.jl package. This python code is available on GitHub (https://github.com/SiFTW/CSV2JuliaDiffEq).
Two different collections of these CSV files were used in this manuscript. One defining the NF-κB, and one defining the model in which TLR, BCR and NF-κB signaling are combined. To assemble each of the two models, the CSV2Julia (https://github.com/SiFTW/CSV2JuliaDiffEq) command was run with the appropriate model definition CSV files as arguments to the CSV2Julia function.

Solving models
Simulation length, initial conditions, and algorithm used in generating solutions (such as tolerance) are provided in the Jupyter Notebooks https://github.com/SiFTW/NFkBModel). The steady state of each simulation was obtained through an extended simulation from the initial conditions (100,000 minutes), and no species was found to be substantially changing by this time point. The endpoint of this steady state simulation was used as the initial conditions for the time course phase. For conditions where stimulation was required, this was added to the model at the start of the time course (t=0).

Cell-to-cell variability
Cell-to-cell variability was approximated (assuming pre-existing differences in expression and degradation rates) as described previously (2). Table S1 shows the parameters which are distributed within the NF-κB model.

Table S1

Parameter	Value	Description
k1_IkBaDegIKK	0.0014	Degradation rate of IκBα by IKK
k1_IkBbDegIKK	0.0014	Degradation rate of IκBβ by IKK
k1_IkBeDegIKK	0.0014	Degradation rate of IκBε by IKK
k1_IkBDeg	0.00024	Degradation rate of IκB in IκB:NF-κB complexes
k1_NFkBDeg	0.00024	Degradation rate of NF-κB dimers in IκB:NF-κB complexes
k1_tIkBb	0.0012	Constant rate of IκBβ gene transcription
kmax_IkBaSynth	0.0048	Kmax for IκBα gene transcription, RelA dimer dependent
kmax_IkBeSynth	7.20E-05	Kmax for IκBε gene transcription, RelA dimer dependent
k1_tIkBaDeg	0.029	Degradation rate for IκBα transcript
k1_tIkBbDeg	0.0029	Degradation rate for IκBβ transcript
k1_tIkBeDeg	0.0038	Degradation rate for IκBε transcript
k1_IkBadeg	0.12	Degradation rate for IκBα
k1_IkBbdeg	0.12	Degradation rate for IκBβ
k1_IkBedeg	0.01155	Degradation rate for IκBε
k1_IkBddeg	0.003	Degradation rate for IκBδ
k1_RelASynth	1.15E-04	Constant rate of RelA gene transcription
basal_p50Synth	1.75E-05	Constant basal rate of p50 gene transcription
basal_RelBSynth	5.14E-05	Constant basal rate of RelB gene transcription
basal_cRelSynth	3.60E-05	Constant basal rate of cRel gene transcription
k1_tRelAdeg	0.0029	Degradation rate for RelA transcript

k1_tp50deg	0.0029	Degradation rate for p50 transcript
k1_tRelBdeg	0.0029	Degradation rate for RelB transcript
k1_tp100deg	0.00096	Degradation rate for p100 transcript
k1_tcReldeg	0.00096	Degradation rate for cRel transcript
k1_monDeg	0.0228	Degradation rate for unbound NF-κB monomers and p100
k1_NIKSynth	0.042	Constant basal rate of NIK synthesis
k1_NIKConstDeg	0.0092	Basal rate of NIK degradation
kmax_p100Synth	1.44E-05	Kmax for p100 gene transcription

Increased RelA/cRel and RelB simulations.

To produce the simulations in Figure 1 C-D, the rates of transcription for RelA, cRel, and RelB were multiplied by 10, producing the increased RelA, increased cRel, and increased RelB models respectively. Each of these simulations was simulated to steady state followed by a time course response to increased canonical stimulation beginning at t=0. Other than the canonical stimulation, simulated as an increase in NEMO-IKK, all parameters were kept consistent from the time course-phase in the steady state-phase.

Models from gene expression data

In order to produce cell line-specific models from gene expression data, parameters for the rates of transcription of RelA, cRel, and RelB, p50, p100 were adjusted. U2932 and RIVA-specific models were created by adjusting these parameters. Gene expression data was downloaded from the Gene Expression Omnibus (https://www.ncbi.nlm.nih.gov/geo/query/acc.cgi?acc=GSE103934)(6). The gene expression (in $\log_{10}$ counts per million reads) was standardized per gene, such that the expression of a particular gene across all cell lines has 0 mean and 1 standard deviation (a *z*-distribution). The default parameter for expression of each gene was multiplied by $10^{z\text{-score}}$. As such a gene with average expression would not have its expression scaled ($10^0 = 1$), while a gene with expression 1 standard deviation higher than the average would have 10 fold higher expression ($10^1=10$). The result was two models, adjusted from the published B-cell model, incorporating cell line-specific gene expression.

Models recapitulating NF-κB fingerprints

To fit the NF-κB model to flow cytometry data from the NF-κB fingerprints RelA and RelB transcription rates were manually adjusted (Figure 4). In addition to the models in which only RelA and RelB was adjusted, cell line-specific models were created with elevated basal NEMO-IKK activity by multiplying the level of activity during the steady state phase 100 fold. As this resulted in changes to the levels of RelA and RelB, expression of these parameters was adjusted to compensate for the increase in NEMO activity and recapitulate NF-κB fingerprints (Figure S4).

Creating a model combining NF-κB, TLR and BCR signaling

CSV files encoding the reactions, rate laws, and parameters for the NF-κB model (above), were combined with files encoding published TLR and BCR models (3, 4). An additional linking module was defined as linking reactions, linking parameters, and linking rateLaws (see the moduleDefinitionFiles

https://github.com/SiFTW/NFkBModel). The model was linked through a single added molecular species defined as "totalActiveIKK", which was defined as the sum of active NEMO-IKK species from the TLR model and the BCR model. As both these models included different abundances of NEMO-IKK, a scaling factor was applied to ensure both modules equally contribute to "totalActiveIKK" and therefore the NF-κB model. Modules (TLR, BCR, NF-κB, and linking) were combined using a bespoke Julia script (combineModels.jl, https://github.com/SiFTW/NFkBModel) to create a single set of CSV files that represent the combined model.

Supplemental References

1. Frazzi R, Cusenza VY, Pistoni M, Canovi L, Cascione L, Bertoni F, et al. KLF4, DAPK1 and SPG20 promoter methylation is not affected by DNMT1 silencing and hypomethylating drugs in lymphoma cells. Oncology reports. 2022;47(1):1-12.
2. Mitchell S, Roy K, Zangle TA, Hoffmann A. Nongenetic origins of cell-to-cell variability in B lymphocyte proliferation. Proceedings of the National Academy of Sciences. 2018;115(12):E2888-E97.
3. Cheng Z, Taylor B, Ourthiague DR, Hoffmann A. Distinct single-cell signaling characteristics are conferred by the MyD88 and TRIF pathways during TLR4 activation. Science signaling. 2015;8(385):ra69-ra.
4. Inoue K, Shinohara H, Behar M, Yumoto N, Tanaka G, Hoffmann A, et al. Oscillation dynamics underlie functional switching of NF-κB for B-cell activation. NPJ systems biology and applications. 2016;2(1):1-9.
5. Mitchell S, Tsui R, Tan ZC, Pack A, Hoffmann A. The NF-κB multidimer system model: A knowledge base to explore diverse biological contexts. Science Signaling. 2023;16(776):eabo2838.
6. Compagno M, Lim WK, Grunn A, Nandula SV, Brahmachary M, Shen Q, et al. Mutations of multiple genes cause deregulation of NF-kappaB in diffuse large B-cell lymphoma. Nature. 2009;459(7247):717-21.
